# Summary of worldwide pediatric malignancies reported after exposure to etanercept

**DOI:** 10.1186/1546-0096-8-18

**Published:** 2010-06-14

**Authors:** Peter McCroskery, Carol A Wallace, Daniel J Lovell, Scott Stryker, Nataliya Chernyukhin, Consuelo Blosch, Debra J Zack

**Affiliations:** 1 Amgen Inc., Thousand Oaks, CA, Seattle WA, and South San Francisco, CA, USA; 2 University of Washington and Seattle Children's Hospital, Seattle, WA, USA; 3 Cincinnati Children's Hospital Medical Center, Cincinnati, OH, USA

## Abstract

**Background:**

Concerns have been raised about a potential link between the use of TNF inhibitors and development of malignancy in the pediatric population. We examined the worldwide experience of etanercept use in pediatric patients and the occurrence of malignancies as reported from clinical trials, registry studies, post-marketing surveillance, and published scientific literature.

**Methods:**

All reports of "malignancy" in pediatric patients (including subjects who received etanercept before age 18 and developed a malignancy before age 22) were collected from the etanercept clinical trials database and global safety database using the Medical Dictionary for Regulatory Activities (MedDRA; v12.0) standardized MedDRA query "Malignancies" from 1998 to August 2009. Cases were collected irrespective of treatment indication. All cases were included regardless of exposure to other TNF blockers or other biologics and whether the other exposure was before or after etanercept.

**Results:**

A total of 18 potential malignancies were identified: 4 leukemias, 7 lymphomas, and 7 solid tumors. Three of the 18 malignancies remain unconfirmed. No malignancies were reported from clinical trials or the open-label extension studies in any indication in children.

**Conclusion:**

The data suggest that there does not appear to be an increased risk of malignancy overall with the use of etanercept. Among etanercept-exposed patients aged 4 to 17 years, the estimated worldwide and US reporting rates for lymphoma were approximately 0.01 per 100 patient-years (1 in 10,000 pt-yrs). While the reported rate of lymphoma is higher in pediatric patients treated with etanercept than in normal children, the expected rate of lymphoma in biologic naïve JIA patients is currently unknown. The risk of TNF inhibitors in the development of malignancies in children and adolescents is difficult to assess because of the rarity of malignant events, the absence of knowledge of underlying frequency of leukemia and lymphoma in JIA, and the confounding use of concomitant immunosuppressive medications.

## Background

Tumor necrosis factor (TNF) is a pro-inflammatory cytokine that plays a role in host defenses and appears in excess in various inflammatory conditions. TNF has been suggested to play a major role in apoptosis and as an antineoplastic in the immunologic response to tumor cells. However, TNF has been shown in other experiments to promote proliferation, invasion, and metastasis of tumor cells [1,2]. Consequently, there is a theoretical but uncertain effect of TNF inhibition on malignancy. Anti-TNF therapies are used to treat several pediatric diseases including juvenile idiopathic arthritis (JIA), inflammatory bowel disease (IBD) and psoriasis (PsO).

JIA is a broad term encompassing a heterogeneous group of persistent inflammatory arthritides in children, including psoriatic arthritis and ankylosing spondylitis [3]. The course of these diseases is variable with some patients developing a progressive destructive arthritis and others experiencing a milder course.

JIA has traditionally been treated with anti-inflammatory medications such as NSAIDs, corticosteroids, methotrexate, and other standard disease modifying agents. More recently, biologic therapies such as tumor necrosis factor (TNF) inhibitors have been added to the treatment armamentarium with dramatic improvement in both the signs and symptoms of JIA as well as outcomes [4-9].

However, concerns have been raised recently about a potential link between the use of TNF inhibitors and the development of malignancy [10-12]. Conflicting reports have appeared in the literature regarding the influence of TNF inhibitors on rates of lymphomas and solid malignancies in adults [1,13-18]. In addition, several case reports have suggested the use of TNF inhibitors may be associated with development of malignancies in children [19,20]. The difficulty in the interpretation of the data stems, in large part, from the low number of JIA patients, the rarity of malignant events, and the lack of an appropriate comparator group of untreated JIA patients. Furthermore, at certain age ranges during childhood, there is an increased background risk to develop either leukemia (younger ages) or lymphoma (teen years) [21,22].

One of the most commonly used TNF inhibitors to treat JIA is etanercept, a fully human soluble receptor Fc fusion protein that binds specifically to TNF. Etanercept has been approved since 1999 for the treatment of polyarticular course JIA in children 2 years and older [10]. Here we present the worldwide experience of etanercept use in pediatric patients and the occurrence of potential malignancies as reported from clinical trials, registry studies, and post-marketing surveillance over the 11-year period from 1998 to 2009. The purpose of this communication is to fully disclose all malignant events in pediatric patients in the entire worldwide etanercept safety database in an attempt to provide information for providers and patients' families to use in their decisions about appropriate JIA therapy.

## Methods

All reports of "malignancy" in pediatric patients (defined as patients who received etanercept before age 18 and who developed a malignancy before age 22) were collected from the sponsor companies' (Amgen and Wyeth [Pfizer]) etanercept clinical trial database and global safety database, using the Standardized Medical Dictionary for Regulatory Activities (MedDRA) Queries (SMQ) version 12.0 term "Malignancies" from 1998 to 13 August 2009. The selection of the age groups was at the recommendation of the US Food and Drug Administration (FDA) and was intended to provide a follow-up observation period of at least 4 years after initiating etanercept therapy. The search of the global safety database included medically confirmed and unconfirmed, serious and nonserious reports of malignancy where etanercept was identified as a suspect, co-suspect, or suspect interacting medication. Cases were collected irrespective of treatment indication and whether treatment was for an approved or unapproved use. All cases were retrieved independently of the reported relationship to causality, and all cases were included regardless of exposure to other TNF inhibitors or other biologics and regardless of whether the exposure to these other agents occurred before or after etanercept. The etanercept global safety database comprises reports from "all sources", including clinical studies (serious adverse events only), open-label extension studies, registry studies, and spontaneous reports (including those from consumers, healthcare professionals, published scientific literature, other companies, and health authorities; and consumer-solicited reports). The published scientific literature is searched weekly using a standardized broad search strategy including "etanercept" and adverse event terms, across bibliographic databases that include ADIS Reactions, BIOSIS, EMBASE and MEDLINE. Individual case reports are identified involving any adverse events associated with etanercept use. Identified cases are entered into the global safety database regardless of the disease state or age of the patient.

All cases were reviewed to identify those with a reported preferred term indicating malignancy. Cases with the following preferred terms were not considered as reports of malignancy and were excluded: bladder cyst, breast cyst, cyst, dermal cyst, fibrous histiocytoma, hemorrhagic ovarian cyst, histiocytosis hematophagic, marrow hyperplasia, melanocytic nevus, myelodysplastic syndrome, neoplasm, neoplasm skin, ovarian cyst, ovarian cyst ruptured, skin papilloma, and synovial cyst. The terms "neoplasm" and "neoplasm skin" were not considered as they are defined as indicating a lesion that is a new growth but do not necessarily imply malignancy. Patients in whom events occur corresponding to these 2 terms and who in fact have a malignancy would be identified by the description of the specific type of malignancy. Cases where the start of etanercept exposure occurred after the age of 18 were not included, with the exception of a single case where etanercept was initiated in a patient aged 18 years and 3 months. Following medical review, cases were grouped first by type of malignancy (leukemia, lymphoma, solid tumor) and then by the age of the patient at the time of diagnosis.

Etanercept exposure was estimated by using the overall commercial market patient-years of exposure for the US and world and applying the proportion of all etanercept use estimated to fall in the age-groups 4 to 11; 12 to 17, and 18 to 22 years. These age-specific proportions were derived from US data from 2 sources: a user survey covering 1998-2002 and a commercial prescription database for the time period from 2005-2009. Calculated estimated US reporting rates of pediatric malignancies with etanercept were compared with US incidence rates for all childhood malignancies as estimated by the Surveillance, Epidemiology, and End Results (SEER) Program of the National Cancer Institute [23], which collects and reports cancer incidence and survival data from cancer registries covering approximately a quarter of the US population, including children.

## Results

No malignancies have been reported to date in any of the randomized controlled clinical trials or open-label extension trials of etanercept in pediatric patients with JIA or psoriasis, which represents 725 patients and 1826 patient-years of exposure as of December 2009. In the worldwide post-marketing and registry experience from 1998 to August of 2009, a total of 18 potential cases of malignancies in pediatric or young adult patients with etanercept exposure were identified and are described in Table 1[5,20,24]. Identified malignancies included 4 cases of leukemia, 7 cases of lymphoma, and 7 cases of solid tumors among patients ranging in age from 4 to 21 years. In 3 cases (Cases 1, 13, and 16), the reported malignancies could neither be confirmed nor ruled out because of a lack of medical history, relevant confirmatory test results, and treatment details. Similarly, in Case 4, the diagnosis of lymphoma remains unconfirmed; test results and biopsies are consistent with acute myelocytic leukemia only.

**Table 1:** Pediatric Malignancy Cases Observed in the Worldwide Safety Database from 1998 to August 13, 2009

Case	Age at malignancy diagnosis/Sex	Preferred term	Country	Length of exposure to ETN	Indication	Confounding medications	Additional comments
1	10 M	Leukemia	US	4 months	Polyarthritis	Methylprednisolone	Malignancy unconfirmed

2	7 M	Acute lymphocytic leukemia	DE	"a few months"	Polyarthritis	MTX - 1 yr	History of splenomegaly prior to starting etanercept

3	4 M	Acute lymphocytic leukemia	DE	8 doses over 3 mos	JIA (Still's)	MTX - 6 mos Prednisolone	Family history of cancer

4	19 M	Acute myeloid leukemia, lymphoma	US	1.5 years	Ankylosing spondylitis	Prednisone	Lymphoma remains unconfirmed Family history of cancer

5	9 M	Hodgkin's lymphoma, Stage IV B	US	ETN for 1 yr; D/C'd	JIA diagnosed at 11 mo of age	MTX - 4 yrs, Cyclosporine - 6 mo, Mycophenolate mofetil - 7 mo, Infliximab - 3.5 yrs	Reported in [1]

6	10 F	Hodgkin's lymphoma stage2a	US	ETN for 1 yr at age 2	JIA diagnosed at 19 mo old	MTX - 3.7 yrs, Infliximab - 2 doses, Leflunomide - 18 mo, Kineret - 1 mo, IV cyclophosphamide - 5 mos, Thalidomide - 7 mo, Rituximab - 2 doses, Adalimumab - 2.4 yrs	Reported in [1]

7	14 M	Diffuse large Bcell lymphoma	DE	7 years	JIA	MTX	

8	15 F	Hodgkin's lymphoma, stage 2	US	4 years	JIA	MTX	Reported in [1]

9	18 F	Bcell lymphoma	GB	3 years	JIA	MTX, azathioprine, cyclosporine	

10	16 F	Lymphomatoid papulosis (lymphoma)	DE	20 months from 7/2004 to 3/2006	Vasculitis diagnosed in 3/94 at age 1.5 yr	MTX 7/98-12/04, Cyclophosphamide 6/07 - 7/07, Azathioprine 9/07 - 11/07, Adalimumab 3/06 - 5/07, Infliximab 5/07 - 7/07, Rituximab 2/08- 3/08, Abatacept 10/08 - 11/08, Prednisolone	Lymphomatoid papulosis by biopsy in 2002; confirmed in 12/08 Lymphoma diagnosed 2/09, unknown type secondary to steroid therapy Off label use for "vasculitis"

11	21 F	Hodgkin's lymphoma, stage 2a	US	3.5 years	JIA diagnosed at age 14	MTX - 6 yrs up to 25 mg/wk	Reported in [2]

12	16 M	Yolk sac tumor	DE	29 days	JIA	MTX - 5 mo Prednisone	

13	17	Bladder cancer	US	NR	NR	No medical information	Malignancy unconfirmed

14	18 F	Papillary thyroid cancer	US	4 years	JIA diagnosed at age 10		

15	18 M	Thyroid cancer	DE	11 months	Spondylo-arthropathy	MTX	Reported in [3].

16	18 F	Thyroid nodule (thyroid neoplasm)	GB	6 months	NR but subject in JIA registry	MTX	Thyroid nodule, malignancy unconfirmed

17	19 F	Malignant melanoma	GB	11 months	Psoriatic arthritis diagnosed age 10	MTX	

18	18 F	Malignant melanoma	GB	11 months	Psoriatic arthritis, JIA		Pregnant; pre-existing pigmented nevus with change noted during first trimester of pregnancy

DE = Germany; GB = Great Britain; JIA = Juvenile idiopathic arthritis; MTX = methotrexate NR = Not Reported; US = United States

In the majority of cases, patients had received one or more concomitant medications; methotrexate was most common (Table 1). Several patients had received other biologic therapies besides etanercept, and in Cases 5 and 6, patients had received other TNF-inhibitor therapies for more than a year after receiving etanercept. In addition, several patients were treated with a number of immunosuppressants, including azathioprine, cyclosporine, cyclophosphamide, mycophenolate, leflunomide, and corticosteroids. Some of these agents have been associated with increased risk of malignancy, especially lymphomas [25,26].

An additional 4 cases were retrieved in the initial search but were later excluded because the malignancy was either pre-existing or subsequently disproven. In one case, a 14-year old had a history of renal cell carcinoma that preceded the use of etanercept therapy for idiopathic pneumonia syndrome. The renal cell carcinoma disease progression continued throughout etanercept treatment. In a second case, acute myeloid leukemia recurrence was reported in a 12-year old. The acute myeloid leukemia preceded the initiation of etanercept therapy. A third case was a report of myelodysplastic syndrome in a 17-year old who had been receiving etanercept for juvenile arthritis for 4 years, but upon medical review the adverse event was considered to be pancytopenia due to myelosuppressive therapy. The patient fully recovered with discontinuation of the DMARD. Finally, follow up with the reporting physician on a case of "possible lymphoma" in a 17-year old revealed that the initially considered diagnosis of lymphoma was never confirmed, and the patient was fully recovered by August 2008.

Worldwide exposure to etanercept among pediatric patients as of July 2009 was estimated at 49,716 patient-years for patients 4 to 17 years old and 33,887 patient-years for patients 18 to 22 years old. Corresponding US exposure was estimated to be 33,409 and 22,349 patient-years, respectively. Reporting rates for malignancies calculated using these exposure estimates are listed in Table 2. By definition, observed case counts in Table 2 for patients with onset of malignancy at age 18 to 22 years did not include cases with exposure after age 18, thus only the reporting rates for the 4-to-17-year-old age group are included for comparison with the incidence rates in normal children. Among etanercept-exposed patients aged 4 to 17 years, the estimated worldwide reporting rate for all malignancies was approximately 0.02 per 100 patient-years and the estimated US reporting rate was slightly less at 0.015 per 100 patient-years. Incidence rates for all malignancies from the US SEER database (2001-2005) are 0.0119 and 0.0169 per 100 person-years for the age groups of 4 to 11 and 12 to 17 years, respectively [23]. The expected malignancy rate for all malignancies in the normal population age group from 4 to 17 years weighted for age distribution is 0.0147 per 100 person-years. The combined rate for all malignancies between those reported for etanercept in this age group and the incidence in normal children are therefore in a similar range at about 1.5 per 10,000 person-years.

**Table 2:** Estimated Reporting Rate of All Malignancies and Lymphoma per 100 Patient-years (PY) of Etanercept Exposure

	4-17 years		18-22 years	
	**Worldwide**	**US**	**Worldwide**	**US**

Estimated exposure to etanercept (PY) through July 2009	49,716	33,409	33,887	22,349

All malignancies (rate/100 PY)	0.020	0.015	0.024*	0.013*

Lymphoma (rate/100 PY)	0.010	0.009	0.006*	0.004*

*Calculated reporting rate for those age 18-22 years includes only cases with exposure prior to age 19 and does not includes cases with first etanercept exposure at age 19-22.

Among etanercept-exposed patients aged 4 to 17 years, the estimated worldwide and US reporting rates for lymphoma were approximately 0.01 per 100 patient-years (1 in 10,000 pt-yrs). Incidence rates for lymphoma in a normal pediatric population from the US SEER database (2001-2005) are lower, but notably increase with increasing age: 0.00147 per 100 person-years for ages 4 to 11 and 0.0035 per 100 person-years for ages 12 to 17 [23]. Weighted for the age distribution, the expected rate in normal children aged 4 to 17 from the SEER database is 0.0026 per 100 pt-yrs (0.26 in 10,000 pt-yrs). The estimated relative risk of lymphoma in etanercept-treated patients in the 4-to-17-year-old age group as compared with the healthy US population is approximately 3.8. The expected rate of lymphoma in untreated pediatric patients with JIA or other inflammatory conditions is unknown.

## Discussion

Concern has been raised about occurrence of malignancies in pediatric patients using TNF inhibitor therapy. In etanercept clinical trials representing 725 patients and 1826 patient-years, there have been no reported cases of malignancy. Here we provide detail on the 18 potential cases of malignancy (15 documented) reported worldwide in pediatric patients receiving etanercept over the years from 1998 to August 2009.

Several factors make it difficult to interpret the results of our findings. For example, assessing causality is often difficult. It is well known that children with certain malignancies, particularly leukemia, may initially present with musculoskeletal symptoms similar to early signs of JIA [27-29]. Studies examining the differential diagnosis of acute lymphoblastic leukemia (ALL) and JIA have revealed that some children presenting with JIA-like symptoms are later diagnosed with ALL, with a time delay of diagnosis ranging from weeks to several months [30,31]. Three of the four leukemia cases reported herein (Cases 1, 2 and 3-all cases of ALL) were diagnosed within 4 months of the patients initiating etanercept therapy and likely represent this overlap in symptomatology before identification of the underlying malignancy.

Secondly, in adults the RA disease itself appears to predispose patients to increased rates of malignancy, particularly lymphoma and leukemia, and patients with the highest disease severity have the most increased risk [15-17,32-35]. Malignancy rates in adult RA patients treated with TNF inhibitors have been evaluated in a number of clinical trials, databases, and registries. The body of available evidence suggests that overall malignancy rates are not increased with TNF inhibitor treatment as compared with an RA population receiving traditional DMARDs alone (ie, no TNF inhibitor) or with the general population. Reported standard incidence ratios for all TNF inhibitors combined have been in the range of 0.94 to 1.1 [36-39]. However, lymphoma risk appears to be higher in adult RA patients using TNF-inhibitors as compared to that of the general population, but appears to be similar or only slightly elevated when compared with a background RA population [15,36-41]. While the safety experience with TNF inhibitors in adult RA patients may provide some insights into the pediatric population, it is important to note that RA and JIA are different diseases and the association of the adult RA disease state with increased risk for lymphoma or leukemia may or may not translate into an increased risk in the JIA population.

The background rates of malignancies in pediatric patients with chronic untreated JIA are not known because malignancies are uncommon in children as a group and because JIA itself is an uncommon disease, although the rate of lymphoproliferative cancers in JIA in the pediatric group has been estimated recently to be 3.8 times the rate in healthy children [42]. Long-term patient registries adequate to measure the risk do not exist. It is possible that chronic active inflammation in children with JIA might also predispose to increased rates of malignancy as suggested in adults.

Thirdly, most JIA patients are not left untreated and so they receive concomitant therapies such as MTX or other immunosuppressives (eg, azathioprine, cyclosporine, cyclophosphamide) that have been associated with increased development of malignancies [25,26]. Development of Hodgkin's lymphoma and other malignancies in adults with RA and children with JIA receiving MTX has been described in several published case reports [19,43,44], but a systematic evaluation of the risk of malignancy in pediatric patients with JIA receiving MTX is not available. Similarly, there is no accurate way to assign causality in cases where many different therapies have been used over time, including multiple DMARDs and biologic agents (see, for example, Cases 5, 6, 9, and 10).

Finally, 4 of the 18 cases identified are questionable as 3 did not have enough information in the post-marketing report to be able to confirm or reject the case (Cases 1, 13, and 16), and 1 other had the diagnosis of malignancy made within 1 month of onset of therapy (Case 12). These cases were still included in this analysis and in reporting rate estimates.

In addition to describing the reported cases, we used estimated etanercept exposure rates to calculate estimated reporting rates for malignancies. Information on the age distribution of estimated etanercept exposure was limited to U.S. information, and the proportion of exposure in the youngest age groups was small; therefore, the estimates can only be considered approximations. Other known limitations of post-marketing surveillance data include underreporting or stimulated reporting, surveillance bias, case ascertainment, and case validation. Thus, these reporting rates do not represent occurrence rates or estimates of incidence rates based on observation time. Occurrence rates are best obtained from clinical or epidemiological studies or population registries such as SEER where both exposure time and event number can be more accurately ascertained. Because of these constraints, the best estimate for malignancy rates in children from this study is derived from the US data for the 4-to-17-year age group. We observed 5 US cases of malignancy in 33,409 patient-years in the 4-to-17-year age group in our analysis (Cases 1, 5, 6, 8, and 13; 1 confirmed lymphoma case, 2 confirmed but highly confounded lymphoma cases, and 2 unconfirmed cases [leukemia and bladder cancer]). This is consistent with the expected total of about 5 US malignancies in this age group based on age-specific data from the US SEER database using a similarly sized population of normal children and a similar number of years of follow-up. Utilizing similar data for rates of lymphoma in general populations of children of these ages, approximately 1 lymphoma case would have been expected whereas our study found 3 US cases in the population of JIA. As discussed above, the background rates of lymphoma in untreated JIA populations are not known. Caution should be exercised in these comparisons because of the limitations of post-marketing surveillance data as listed above.

None of the identified malignancies with etanercept occurred in Crohn's disease patients, most likely because etanercept is not approved for this indication. Other TNF inhibitors are approved for use in this population, however, and malignancies associated with TNF inhibitor use in pediatric patients with Crohn's disease have been reported [45,46]. Factors involved in the development of malignancy in patients with Crohn's disease may differ from those in patients with JIA since children with chronic severe Crohn's disease have a risk of developing hepatosplenic T cell lymphoma [46]. Because the children with the most severe cases of either JIA or Crohn's disease are also the children most likely to be treated with anti-TNF therapy, it is difficult to make a determination of causality.

## Conclusions

In summary, we identified 18 potential malignancies worldwide in pediatric and young adult patients receiving etanercept over 11 years of experience. The development of a malignancy following etanercept is rare, roughly 1.5 cases in 10,000 pt-yrs. The data suggest that there does not appear to be an increased risk of malignancy overall with the use of etanercept in children. While the reported rate of lymphoma is higher in pediatric patients treated with etanercept than in normal children, the expected rate of lymphoma in JIA patients is currently unknown, although a recent study notes that there is a significantly elevated risk of incident lymphoproliferative cancers in biologic-naïve JIA patients. In addition, the risk of etanercept use in the development of malignancies in children and adolescents is difficult to assess because of the low incidence and prevalence of JIA, the rarity of malignant events in children, the ability of some forms of malignancy to present with symptoms mimicking JIA, the absence of knowledge of the underlying frequencies of specific malignancies in untreated JIA, and the confounding use of concomitant immunosuppressive medications.

## Abbreviations

ALL: acute lymphocytic leukemia; DE: Germany; DMARD: disease-modifying anti-rheumatic drug; FDA: Food and Drug Administration; GB: Great Britain; JIA: juvenile idiopathic arthritis; MedDRA: Medical Dictionary for Regulatory Activities; MTX: methotrexate; NR: not reported; NSAID: nonsteroidal anti-inflammatory drug; PY: patient-year; RA: rheumatoid arthritis; SEER: Surveillance, Epidemiology, and End Results (program); SMQ: standardized MedDRA query; TNF: tumor necrosis factor; US: United States;

## Competing interests

C. Wallace reports receiving research grants from Amgen, Centocor and Pfizer. D. Lovell reports receiving research grants and/or consulting or Speaker fees from Abbot, Amgen, Bristol-Meyers Squibb, Centocor, Hoffmann-La Roche Inc, Novartis, Inc, Pfizer, Regeneron, UBC, Wyeth Pharmaceuticals, and Xoma, Inc. P. McCroskery, S. Stryker, N. Chernyukhin, C. Blosch, and D.J. Zack are full-time employees of Amgen Inc and own stock or stock options in Amgen Inc.

## Authors' contributions

PM and DJZ conceived the analysis; PM, SS, NC, and CB conducted the database searches; all authors contributed to the analysis and interpretation of the data. The initial manuscript draft was written with the help of a medical writer based on guidance from PM, DJZ, CAW, and DJL; all authors critically reviewed and revised drafts; and all authors approved the final version.
